# Geochemical Modeling of Heavy Metal Removal from Acid Mine Drainage in an Ethanol-Supplemented Sulfate-Reducing Column Test

**DOI:** 10.3390/ma16030928

**Published:** 2023-01-18

**Authors:** Keishi Oyama, Kentaro Hayashi, Yusei Masaki, Takaya Hamai, Shigeshi Fuchida, Yutaro Takaya, Chiharu Tokoro

**Affiliations:** 1 Faculty of Science and Engineering, Waseda University, 3-4-1 Okubo, Shinjuku-ku, Tokyo 169-8555, Japan; 2 Japan Organization for Metals and Energy Security (JOGMEC), 2-10-1 Toranomon, Minato-ku, Tokyo 105-0001, Japan; 3 Department of Marine Resources and Energy, Tokyo University of Marine Science and Technology, 4-5-7 Konan, Minato-ku, Tokyo 108-8477, Japan; 4 Faculty of Engineering, The University of Tokyo, 7-3-1 Hongo, Bunkyo-ku, Tokyo 113-8656, Japan

**Keywords:** acid mine drainage (AMD), sulfate-reducing bacteria (SRB), passive treatment (PT), heavy metal removal, geochemical modeling

## Abstract

A passive treatment process using sulfate-reducing bacteria (SRB) is known to be effective in removing heavy metals from acid mine drainage (AMD), though there has been little discussion of the mechanism involved to date. In this work, a sulfate-reducing column test was carried out using supplementary ethanol as an electron donor for microorganisms, and the reaction mechanism was examined using geochemical modeling and X-ray absorption fine structure (XAFS) analysis. The results showed that Cu was readily removed from the AMD on the top surface of the column (0–0.2 m), while Zn and Cd depletion was initiated in the middle of the column (0.2–0.4 m), where sulfide formation by SRB became noticeable. Calculations by a developed geochemical model suggested that ethanol decomposition by aerobic microbes contributed to the reduction of Cu, while sulfide produced by SRB was the major cause of Zn and Cd removal. XAFS analysis of column residue detected ZnS, ZnSO_4_ (ZnS oxidized by atmospheric exposure during the drying process), and CuCO_3_, thus confirming the validity of the developed geochemical model. Based on these results, the application of the constructed geochemical model to AMD treatment with SRB could be a useful approach in predicting the behavior of heavy metal removal.

## 1. Introduction

Passive treatment (PT) systems using various natural biological and geochemical reactions are widely recognized as a promising technique to treat acid mine drainage (AMD) containing toxic elements such as manganese (Mn), iron (Fe), copper (Cu), zinc (Zn), arsenic (As), cadmium (Cd), and lead (Pb). Such systems are considered advantageous in terms of environmental impact, operation cost, and electrical power requirements, compared with active treatment systems, which require the continuous addition of neutralizing agents such as slaked or quick limes [1,2]. A variety of PT systems have thus been developed over the decades, such as oxic/anoxic limestone channels and wetlands [2,3,4], and the appropriate system needs to be chosen based on the quality and quantity of the targeted AMD.

To enhance the efficiency of PT systems, microbiological activities are spontaneously and/or artificially incorporated into the reaction process. Among various microbiological candidates such as Fe-oxidizing/-reducing [5] and Mn-oxidizing microorganisms [6], sulfate-reducing bacteria (SRB) are employed especially for the reduction of toxic divalent metals such as Cu, Zn, Pb, and Cd. They provide sulfide ions via the microbiological reduction of sulfate ions abundantly present in AMD, enabling the formation of metal sulfide precipitates at circumneutral pH [7]. Due to the high affinity between sulfides and such divalent metals, the stable and immediate immobilization of toxic metals is easily achievable even if the concentrations of target metals are trace [8].

For the maintenance of a PT process in which SRBs are employed, organic carbons need to be supplemented for microbiological heterotrophic growth [9,10] because of the scarce amount of soluble organic carbon originally present in AMD (<10 mg/L) [11]. A variety of organic carbon sources have been assessed as electron donors for SRB growth, such as methanol [12,13,14], ethanol [15,16,17], lactate [16,18], glucose [17], and cellulose [19] as simple/direct organic carbons, and mushroom compost [20], sheep manure [19], liquid/solid whey [17], rice husk [21], and molasses [14] as complex/indirect organic carbons. The latter type of carbon has several advantages: (i) the consistent, long-term supply of low-molecular carbon into the system and (ii) the maturation of the microbiological population structure by simultaneously dissolving several kinds of low-molecular carbon. However, such high-molecular carbon needs to be decomposed by other microbes (e.g., fermentative microorganisms) prior to metabolism by SRB for their use as an energy source [22]. This leads to the reaction mechanism in the system becoming excessively complex. On the other hand, the former type of carbon simplifies it; microorganisms other than SRB are less involved in the decomposition of carbons. Hence, simple organic carbon is thought to be more appropriate for the assessment of the reaction mechanisms involved in the SRB-employed PT process.

To better understand these reaction mechanisms, previous studies have attempted to geochemically model a PT process in which SRBs are employed. In order to reproduce their metabolism, a series of kinetic equations have been obtained via data-fitting with empirical results [23,24,25]. Additionally, in terms of precipitate formation induced by SRB metabolism, researchers generally have taken thermodynamic chemical equilibrium into account. VMINTEQ software (ver. 3.1) has often been used to calculate the saturation index (SI) and estimate the species of precipitates [19,26,27]. However, these are limited studies spontaneously considering both the microbiological kinetic reaction and chemical equilibrium of mineral formation in one model, thus failing to estimate the metal removal behavior from AMD quantitively. Furthermore, the chemistry of sulfate and sulfide has been the main subject of discussion in these studies, while less attention has been paid to the decomposition of ethanol and the resultant carbonate formation. Kaksonen et al. [28] used a fluidized-bed reactor with ethanol as an energy source for SRB and found that acetate oxidation is the rate-limiting step of sulfidogenic ethanol oxidation. However, that study also lacks a discussion of heavy metal precipitation resulting from microbiologically induced carbonate production. Overall, for an accurate estimation of an SRB-employed PT system by geochemical modeling, more comprehensive information, including microbiological kinetic and chemical equilibrium reactions, need to be accumulated.

In this study, we thus aimed to investigate the underlying reaction mechanism involved in an SRB column test, especially in terms of heavy metal removal from AMD. Based on the results of the column test, we constructed a geochemical model incorporating the kinetics reaction of microbiological metabolism, the chemical equilibrium of ionic and precipitation species, and one-dimensional advection in the column. A further detailed mechanism was then discussed through a comparison between the experimental results and the constructed geochemical model. XAFS analysis was also carried out to identify the amorphous secondary minerals formed during the experiment, which would be beneficial to validate the constructed geochemical model.

## 2. Materials and Methods

### 2.1. Sulfate-Reducing Column Test

Prior to the column test, the AMD solution obtained at the A mine site in Japan was neutralized to pH 7.0 by adding limestone to precipitate and remove all soluble Fe and Al. The chemical composition of the neutralized AMD used for the subsequent SRB column test is listed in Table 1.

**Figure 1:**
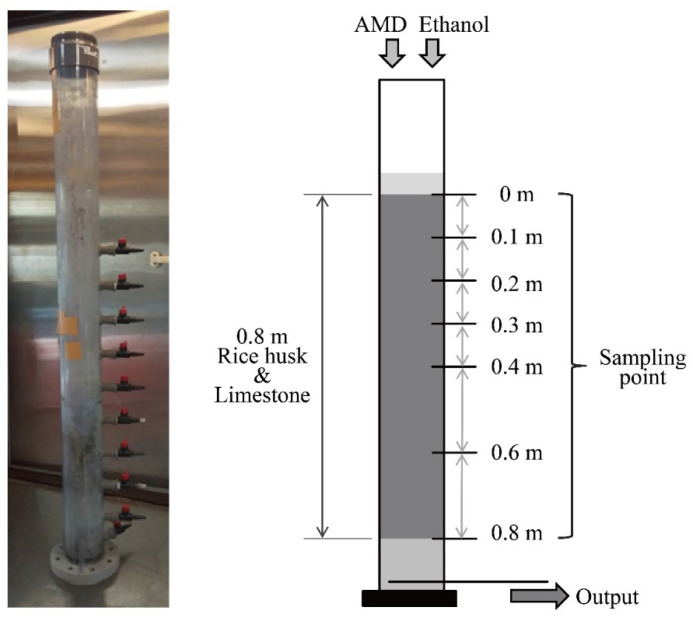
A picture and configuration of the sulfate-reducing column reactor.

The configuration of the sulfate-reducing column used in this study is shown in Figure 1. The laboratory-scale down-flow column reactor (internal diameter 0.1 m) was assembled using polyvinyl chloride, with 7 sampling ports at different column heights: 0, 0.1, 0.2, 0.3, 0.4, 0.6, and 0.8 m from the top. This column was filled with a mixture of 0.7 kg rice husks (support material for microorganisms) and 2.8 kg limestone (20–40 mm in particle size) so as to set approximately 0.8 m in thickness. Neutralized AMD was fed from the top of the column at a flow rate of 2.2 mL/min; hydraulic retention time (HRT) was calculated to be 25 h. At the same time, a supplemental ethanol solution with a concentration of 11.3 mmol/L (FUJIFILM Wako Pure Chemical Corp., Osaka, Japan) was added to the column as an electron donor for sulfate-reducing microorganisms at a flow rate of 0.2 mL/min; the final concentration of ethanol in the input solution was expected to be 1.0 mmol/L. This test was operated in duplicate under temperature-controlled conditions at 15 °C.

### 2.2. Sampling and Solution Analysis

After the initiation of the column test on August 23rd, 2019, solution samples were routinely taken twice a week, from each sampling port, for a period of 85 days to monitor pH and DO levels using MM-43X (TOA DKK) and HQ30d (HACH), respectively. These samples were filtered through a 0.45 μm membrane filter (mixed cellulose ester; Advantec) and then used to determine the concentrations of Cu, Zn, and Cd by ICP-OES (Agilent 5110 ICP-OES, Agilent Technologies Inc., Santa Clara, CA, USA). Concentrations of sulfate (SO_4_^2−^) and acetate in the filtrate were quantified by ion chromatography (Dionex ICS-6000, Thermo Fisher Scientific Inc., Waltham, MA, USA). The concentration of sulfide (HS^−^) was measured by the methylene blue method [29]. The concentration of ethanol was determined by UPLC equipped with an ion exclusion column (IC-Pak Ion Exclusion Column 7 µm, 7.8 mm × 300 mm; Waters Corp., Milford, MA, USA). Total inorganic carbon (TIC) was quantified by a TOC analyzer (TOC-L, Shimadzu Corp., Kyoto, Japan). The detection limits for each chemical specie were as follows: Cu 0.18 (µg/L); Zn 0.08 (µg/L); Cd 0.06 (µg/L).

### 2.3. Geochemical Modeling by PHREEQC

In this study, the geochemical code PHREEQC (ver.3, USGS) was used, which is commonly employed for the geochemical modeling of wastewater treatment systems [30,31]. This modeling enables us to simulate the behaviors of chemical species in the sulfate-reducing column reactor, considering the chemical equilibrium, kinetic reactions, and one-dimensional advection. In this modeling, the elements and their ionic or precipitation species listed in Tables S1 and S2 were taken into account for calculation purposes.

Since the effect of ionic diffusion was assumed to be negligible, compared with the effect of advection, the model calculation was performed using the general advection equation (Equation (1)) [32]:

*dC*/*dt* = −*v* (*dC*/*dx*) − (*dq*/*dt*) (1)

where *C* is the concentration of chemical species (mol/L), *t* is the residence time (sec), *v* is the pore water flow velocity (m/s), *x* is the column distance (m), and *q* is the concentration in the solid phase (expressed as mol/L in the pores). The Dirichlet boundary condition was employed for calculations as follows:

*C*(*x*_end_, *t*) = *C*_0_ (2)

Table S3 shows the parameters used for advection analysis. The flow velocity was calculated from the HRT.

### 2.4. XAFS Analysis of SRB Column-Packing Residue

To identify the heavy metal-containing amorphous secondary minerals formed in the column, XAFS analysis using synchrotron X-rays was performed. Solid column-packing residue at each depth was taken out at the end of the test and dried in an oven at 50 °C for 12 h. The dried samples were then used for analyses of Zn K-edge (9659 eV), Cu K-edge (8979 eV), and Cd K-edge (26,711 eV) at the BL11S2 beamline in the Aichi Synchrotron Radiation Center. Fluorescence mode using silica (111) monochromator crystals was chosen to obtain XAFS spectra; only the X-ray absorption near edge structure (XANES) region was targeted for the measurement. Obtained spectra were then analyzed using Athena software packages (ver. 0.9.26) provided by Demeter [33]. The spectra of standard samples were superimposed on the experimental spectra by linear combination fitting (LCF) to determine the fraction of the secondary minerals formed in the column-packing residue.

## 3. Results and Discussion

### 3.1. Time-Dependent Performance of Sulfate-Reducing Column

**Figure 2:**
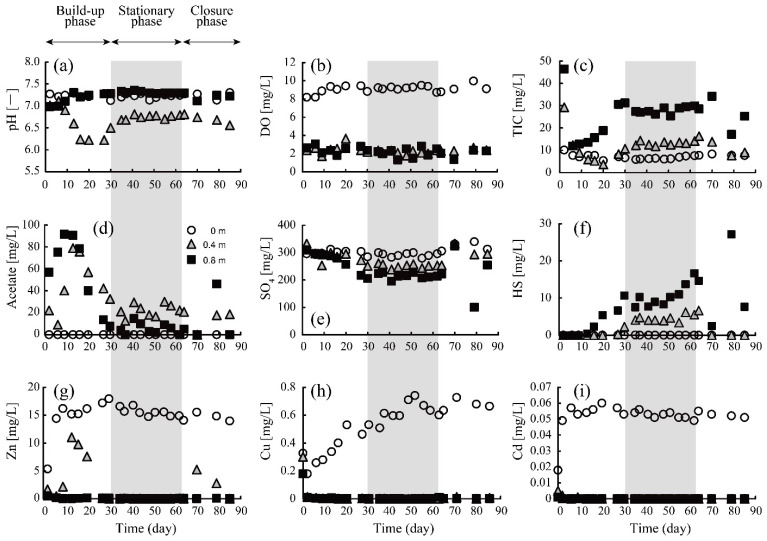
Chronological changes in pH (**a**), and concentrations of DO (**b**), TIC (**c**), acetate (**d**), sulfate (**e**), sulfide (**f**), Zn (**g**), Cu (**h**), and Cd (**i**) at depths of 0 m (top surface of the column), 0.4 m, and 0.8 m.

Figure 2 shows the change in parameters as a function of time at depths of 0, 0.4, and 0.8 m, respectively. The duration of the column test was divided into three periods: (i) build-up phase; (ii) stationary phase (shown with a gray square in Figure 2); and (iii) closure phase. In the build-up phase, the microbiota in the column would have yet been well-organized; Sato et al. [34] also reported that the drastic change in the abundance of SRB occurred during the first 30 days of their acclimation period. For this reason, pH varied unstably from 6.2 to 7.3, especially at the middle depth of the column (0.4 m; Figure 2a). The immediate DO depression below 0.4 m right after the initiation of the column test (Figure 2b) indicated DO consumption by aerobic microbiological metabolism in the upper part of the column. Contrarily, sulfate reduction and sulfide production were barely evident until day 15 (Figure 2e,f), suggesting that the SRB had not yet adequately dominated the population. Instead, the robust solubilization of acetate and TIC (=carbonate) was noticeable in this phase (Figure 2c,d), which might be explained by the decomposition of ethanol—mainly by the metabolism of aerobic microbes—according to the following equations:

C_2_H_5_OH + O_2_ → CH_3_COO^−^ + H_2_O + H^+^ (3)

CH_3_COO^−^ + 2O_2_ → 2HCO_3_^−^ + H^+^ (4)

Furthermore, Zn, Cu, and Cd concentrations rapidly dropped to reach almost 0 mg/L at ~0.4 m (Figure 2g–i). Considering the negligible levels of sulfate reduction and sulfide production here (Figure 2e,f), the observed heavy metal removal from the AMD could not be attributed to the formation of metal sulfides but could be explained as the result of carbonate precipitation facilitated by the reaction shown in Equation (4). Most likely, after SRB acclimated to the column environment, the sulfate reduction and following sulfide production became noticeable (~day 15), with an accompanying decline in acetate concentration, which was consumed as the electron donor for SRB (Figure 2d–f).

In the stationary phase, from day 30 to 63, the stable performance of the sulfate-reducing column was maintained. DO was consistently kept below 3.0 mg/L, which would be a sufficiently low level to activate SRB metabolism. In contrast to the build-up phase, acetate concentration was maintained at a moderate level (<40 mg/L), while a large amount of TIC was continuously produced. SRB was shown to successfully utilize the supplemented ethanol and its decomposition product (i.e., acetate) as an electron donor for their metabolism [35], according to Equations (5) and (6):

SO_4_^2−^ + 2C_2_H_5_OH → HS^−^ + 2CH_3_COO^−^ + 2H_2_O + H^+^ (5)

SO_4_^2−^ + CH_3_COO^−^ → HS^−^ + 2HCO_3_^−^ (6)

As a result, constant sulfate reduction and sulfide production were successfully achieved during this phase (Figure 2e,f). Heavy metals were completely removed from the solution even at a depth of 0.4 m (Figure 2g–i) via the formation of either sulfide and/or carbonate precipitates, which will be further discussed in the following sections.

In the closure phase, inlet flow halted due to the breakdown of the pump, causing serious damage to SRB activity. Consequently, further sulfate reduction and sulfide formation were not observed.

### 3.2. Mechanism Discussion on Sulfate-Reducing Column Based on Geochemical Modeling and XAFS Analysis

To assess the reaction mechanism in the column, a geochemical model was developed, and its calculation results were compared to the experimental values obtained from the sulfate-reducing column test. In addition, XAFS data of column residue were compared with the possible precipitates estimated by the model’s calculations to verify the reliability of the constructed geochemical model.

#### 3.2.1. Kinetic Equations Incorporated into the Geochemical Modeling

Chemical equilibrium calculations enable the reproduction of the metal precipitation behavior only but not the expression of the microbiological reactions (e.g., sulfate reduction and carbonate production). This indicates that microbial metabolisms must be incorporated into the model as kinetic equations. Hence, in addition to the chemical equilibrium reactions listed in Tables S1 and S2, the following kinetic reactions are included in the model calculations in order to consider the decomposition of ethanol into acetic acid (Equation (7)) and the decomposition of acetate into carbonate (Equation (8)) by aerobic microorganisms, as well as the decomposition of ethanol into acetate (Equation (9)), the decomposition of acetate into carbonate (Equation (10)), and the reduction of sulfate to sulfide (Equation (11)) by SRB:

−*d*[C_2_H_5_OH]/*dt* = *k*_1_ [C_2_H_5_OH][DO] (*k*_1_ = 2.4 × 10^−1^) (7)

−*d*[CH_3_COOH]/*dt* = *k*_2_[CH_3_COOH][DO] (*k*_2_ = 3.6 × 10^−2^) (8)

−*d*[C_2_H_5_OH]/*dt* = *k*_3_[C_2_H_5_OH][SO_4_^2−^] (*k*_3_ = 4.8 × 10^−2^) (9)

−*d*[CH_3_COOH]/*dt* = *k*_4_[CH_3_COOH][SO_4_^2−^] (*k*_4_ = 9.8 × 10^−3^) (10)

−*d*[SO_4_^2−^]/*dt* = *k*_5_[SO_4_^2−^] (*k*_5_ = 5.0 × 10^−6^) (11)

where [C_2_H_5_OH], [CH_3_COOH], [DO], and [SO_4_^2−^] indicate the concentrations of ethanol, acetate, DO, and sulfate (mg/L), respectively, and *k*_1–5_ indicates the kinetic constant of each equation (*k*_1–4_: L/mg/s, *k*_5_: 1/s). Kinetic constants were fitting parameters via the numerical fitting of the reaction model to the experimental results. Note that the average value of each chemical parameter during the stationary phase (days 30 to 63 in Figure 2) was employed and used as the fitting data. All microbiological reactions shown above (Equations (7)–(11)) were regarded as catalytic reactions throughout the stationary phase.

To consider the dissolution of calcite, the kinetic equation proposed by Plummer et al. [36] was employed, as shown in Equations (12) and (13):

−*d*[Calcite]/*dt* = *k*_6_*A*(1−SI_calcite_)^2/3^ (12)

*k*_6_ = −29.59[H^+^] − 144.9[CO_2_] − 322.9[H_2_O] (at 15 °C) (13)

where [Calcite] is the amount of calcite in the system (g), *k*_6_ is the kinetic constant of calcite dissolution (g/m^2^/s), *A* is the specific surface area of calcite (m^2^/g), SI_calcite_ is the saturation index of calcite, and [H^+^], [CO_2_], and [H_2_O] are the concentrations of each chemical species (g/L).

#### 3.2.2. Comparison between the Geochemical Modeling and Experimental Observations

**Figure 3:**
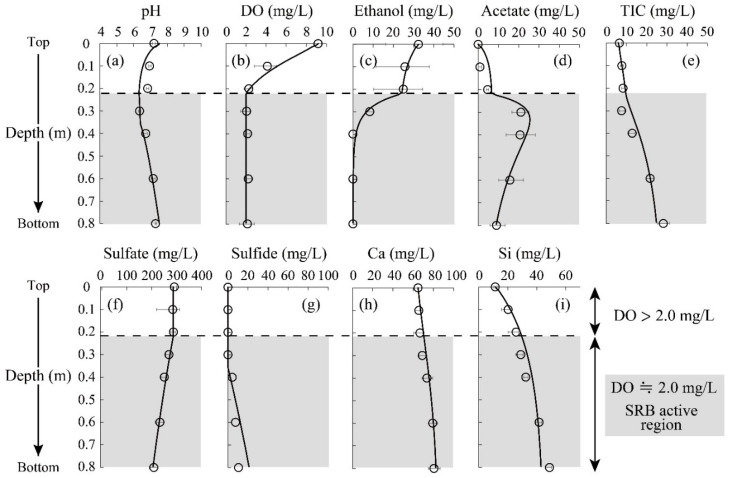
Changes in pH (**a**), and concentrations of DO (**b**), ethanol (**c**), acetate (**d**), TIC (**e**), sulfate (**f**), sulfide (**g**), Ca (**h**), Si (**i**) as a function of column depth. Plots and solid lines indicate the experimental results and geochemical model fitting, respectively. Error bars depict the average values of the duplicate test.

Figure 3 shows the changes in experimental parameters and fitting results with the developed geochemical model as a function of depth. At a shallow column depth, the DO concentration started to decline (Figure 3b). When the DO concentration reached a level of ~2 mg/L at 0.2 m, SRB seemed to be activated, initiating sulfate reduction (Figure 3f), sulfide formation (Figure 3g), ethanol decomposition (Figure 3c), and both acetate and carbonate formation (Figure 3d,e). Sulfate concentration finally reached 214 mg/L at the output of the column (Figure 3f). The reduction efficiency observed here was comparable with the previous report; indigenous SRB was capable of reducing 32% of initially added sulfate [37]. The presence of Cu (>10 mg/L) was reported to show an inhibitory effect on SRB metabolism [38], which was not visible here due to the trace Cu concentration in the AMD solution.

A slight difference was seen between the experimental plots and model fitting in the case of sulfide concentration (Figure 3g), even though most of the other parameters showed great consistency (Figure 3). It was hypothesized that all sulfates were reduced to sulfides by SRB in the model calculation, but, in practice, various intermediates would be formed along the way to biologically transform sulfate into sulfide [39]. Ignorance of the involvement of these intermediates would thus explain the discrepancy between the experimental results and model calculations. At the bottom of the column (0.6–0.8 m), supplemented ethanol was completely converted to acetate and carbonate by SRB (Figure 3c–e), which was greatly fit by the constructed geochemical model. This suggests that the kinetic equations used in this study (i.e., Equations (7)–(11)) reasonably reproduce the metabolism of microorganisms during the SRB column test. Based on the negligible Ca dissolution (Figure 3h), the calcite in this system was assumed to be less reactive due to the relatively high pH of the neutralized AMD solution.

**Figure 4:**
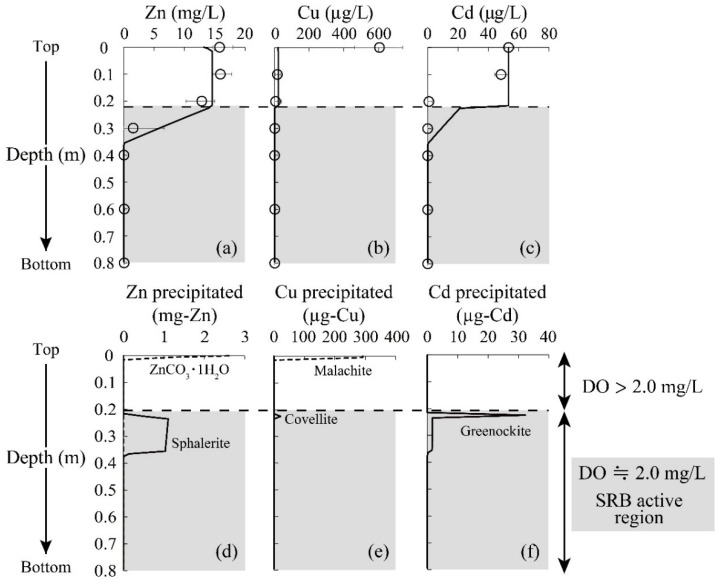
Changes in the heavy metal concentrations (plots) and geochemical model fitting (solid line) as a function of column depth (**a**–**c**). Error bars depict the average values of the duplicate test. Estimated precipitates by model calculation are also shown (**d**–**f**).

In terms of heavy metal reductions, it was seen that the concentrations of Zn and Cd declined at a depth of 0.2–0.4 m to reach almost 0 mg/L (Figure 4a,c). Since these metal depletions were accompanied by the initiation of sulfate reduction and sulfide production (Figure 3f,g), the reduction of Zn and Cd was likely triggered by the formation of sulfide precipitate facilitated by SRB. Indeed, ZnS was shown to be the major precipitate of immobilized Zn based on the geochemical calculation, as shown in Figure 4d; no soluble Zn was seen at greater depths. A slight decrease in Zn concentration was observed at the top of the column, which was due to the formation of carbonate precipitate (i.e., Smithsonite: ZnCO_3_) based on the model calculation (Figure 4d).

**Figure 5:**
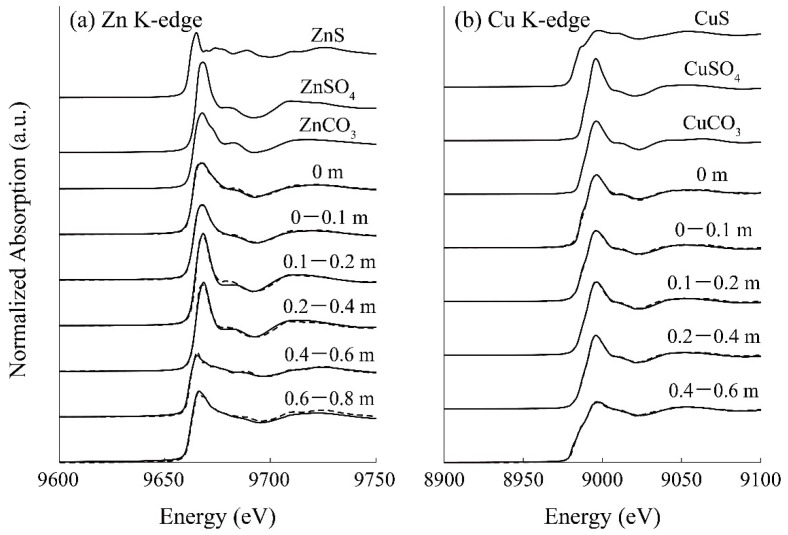
(**a**) Zn K-edge and (**b**) Cu K-edge XANES spectra of reference samples and the solid residue taken from the column at various depths. The dotted lines indicate the LCF fitting results.

Figure 5a shows the Zn K-edge spectra of the reference standards and the column-packing residue taken from each depth, and LCF fitting results against these spectra are summarized in Table 2. A part of the soluble Zn was found immobilized as carbonates at the top surface of the column from the LCF fitting (Table 2), which was consistent with the model calculation (Figure 4d). Moreover, the residue at a depth of 0.2–0.4 m was found to be mainly composed of ZnSO_4_, and its spectrum was expressed with the superimposition of 3% ZnS, 91% ZnSO_4_, and 4% ZnCO_3_ (Table 2). ZnS formed in the column was assumed to be easily oxidized once it was exposed to the air during the drying process, thereby explaining the detection of ZnSO_4_. These observations confirmed the good agreement between the geochemical modeling and solid residue analysis by XAFS.

As for Cu, almost all was readily precipitated after the introduction of AMD into the column (at the top surface of the column; Figure 4b). The model fitting suggested that the Cu carbonate mineral, malachite (Cu_2_CO_3_(OH)_2_), is the dominant precipitate form (Figure 4e), while most other studies insisted that the CuS formation contributed to the Cu reduction [40,41]. The XAFS spectrum near the top surface of the column (0 m) also showed high homology with CuCO_3_ (85%). Carbonate ions were mainly generated via ethanol decomposition by aerobic microorganisms growing in the upper part of the column (Equation (5)), and this was likely to accelerate the removal of Cu from AMD. Unimmobilized Cu at the top of the column was then solidified via covellite (CuS) formation in the middle of the column, where sulfide production by SRB was noticeable. Overall, XAFS analysis verified that the constructed geochemical model effectively reproduced the behaviors of heavy metals removed from AMD in the SRB column.

Unfortunately, the behavior of Cd estimated by the model calculation deviated from the experimental plots; in practice, Cd concentration rapidly declined at a shallower depth (0.2 m; Figure 4c). Since the model’s calculations did not consider the surface complexation of Cd onto secondary minerals formed in the system (e.g., manganese oxide [42]), further refinement of geochemical modeling is needed for higher fitting accuracy.

## 4. Conclusions

An ethanol-supplemented SRB column test was carried out to treat AMD, and its reaction mechanism was discussed using geochemical modeling and XAFS analysis. We successfully developed a geochemical model that effectively reproduced reactions that occurred during the column test. A comparison of model calculations with experimental results allowed us to conclude that the carbonate production by aerobic microorganisms induced the immobilization of Cu as Cu_2_CO_3_(OH)_2_ at the upper part of the column (0–0.2 m), while sulfide production by SRB facilitated the precipitation of Zn as ZnS at the middle of the column (0.2–0.4 m). In addition, the XAFS analysis of column residues indicated that Zn and Cu were removed from the AMD mainly as a sulfate (ZnS oxidized by atmospheric exposure during the drying process) and carbonate, respectively, further verifying the reliability of the constructed geochemical model. On the other hand, the behavior of Cd removal was not reproduced well, which requires the refinement of the model by considering the metal adsorption onto secondary minerals (e.g., the surface complexation of Cd onto the manganese oxides). This constructed model may be beneficial not only for mechanism discussion purposes but also for the prediction of heavy metal reduction behavior when it is applied to an SRB-using AMD treatment process, even with a different configuration. To expand the applicability of the constructed model, further investigation is thought to be necessary in future studies, for example, using other kinds of organic carbon sources (e.g., lactate and propionic acid) and investigating the temperature effects.

## Figures and Tables

**Table 1:** Chemical composition of neutralized AMD used for the column test (average, maximum, and minimum values of 22 samples).

	Neutralized AMD (Influent Water)		
	Average	Max	Min
pH	7.21	7.28	7.12
DO (mg/L)	9.17	9.50	8.72
SO_4_^2−^ (mg/L)	291	300	279
TIC (mg-HCO_3_^−^/L)	32.7	38.5	29.7
Zn (mg/L)	15.8	17.9	14.7
Cu (mg/L)	0.606	0.741	0.463
Cd (mg/L)	0.0533	0.0570	0.0510
Ca (mg/L)	64.8	68.2	61.2
Si (mg/L)	24.3	26.8	22.2
Fe (mg/L)	0.0302	0.0700	0.00690
Al (mg/L)	0.0808	0.144	0.0150

**Table 2:** LCF fitting results against the XAFS spectra of column residues taken at various depths.

Depth (m)	Zn K-Edge				Cu K-Edge			
	Fraction (%)			R-Factor	Fraction (%)			R-Factor
	ZnS	ZnSO_4_	ZnCO_3_		CuS	CuSO_4_	CuCO_3_	
0	19.0	0.79	81.7	0.0023	15.5	0.00	84.5	0.0108
0–0.1	22.6	34.4	43.9	0.0010	23.6	4.73	71.7	0.0038
0.1–0.2	1.82	97.2	0.00	0.0063	16.3	20.6	63.1	0.0032
0.2–0.4	3.18	90.7	3.75	0.0031	25.1	41.9	33.0	0.0023
0.4–0.6	81.4	17.0	1.79	0.0036	81.9	18.1	0.00	0.0019
0.6–0.8	61.3	30.4	13.4	0.0046	N.D.	N.D.	N.D.	N.D.

## Data Availability

Not applicable.

## Supplementary Materials

The following supporting information can be downloaded at: https://www.mdpi.com/article/10.3390/ma16030928/s1.

## References

[1] Ziemkiewicz P.F., Skousen J.G., Simmons J. (2003). Long-term performance of passive acid mine drainage treatment systems. Mine Water Environ..

[2] Skousen J., Zipper C.E., Rose A., Ziemkiewicz P.F., Nairn R., McDonald L.M., Kleinmann R.L. (2017). Review of passive systems for acid mine drainage treatment. Mine Water Environ..

[3] Ziemkiewicz P., Skousen J.G., Brant D., Sterner P., Lovett R. (1997). Acid mine drainage treatment with armored limestone in open limestone channels. J. Environ. Qual..

[4] Alcolea A., Vázquez M., Caparrós A., Ibarra I., García C., Linares R., Rodríguez R. (2012). Heavy metal removal of intermittent acid mine drainage with an open limestone channel. Miner. Eng..

[5] Kirby C., Thomas H., Southam G., Donald R. (1999). Relative contributions of abiotic and biological factors in Fe (II) oxidation in mine drainage. Appl. Geochem..

[6] Hallberg K.B., Johnson D.B. (2005). Biological manganese removal from acid mine drainage in constructed wetlands and prototype bioreactors. Sci. Total Environ..

[7] Xu Y., Chen Y. (2020). Advances in heavy metal removal by sulfate-reducing bacteria. Water Sci. Technol..

[8] Neculita C.M., Zagury G.J., Bussière B. (2007). Passive treatment of acid mine drainage in bioreactors using sulfate-reducing bacteria: Critical review and research needs. J. Environ. Qual..

[9] Rose A.W., Means B., Shah P. Methods for passive removal of manganese from acid mine drainage. Proceedings of the 24th West Virginia Surface Mine Drainage Task Force Symposium.

[10] Nielsen G., Coudert L., Janin A., Blais J.F., Mercier G. (2019). Influence of organic carbon sources on metal removal from mine impacted water using sulfate-reducing bacteria bioreactors in cold climates. Mine Water Environ..

[11] Kolmert Å., Johnson D.B. (2001). Remediation of acidic waste waters using immobilised, acidophilic sulfate-reducing bacteria. J. Chem. Technol. Biotechnol..

[12] Tsukamoto T., Miller G. (1999). Methanol as a carbon source for microbiological treatment of acid mine drainage. Water Res..

[13] Glombitza F. (2001). Treatment of acid lignite mine flooding water by means of microbial sulfate reduction. Waste Manag..

[14] Nielsen G., Janin A., Coudert L., Blais J.F., Mercier G. (2018). Performance of sulfate-reducing passive bioreactors for the removal of Cd and Zn from mine drainage in a cold climate. Mine Water Environ..

[15] Sahinkaya E., Gunes F.M., Ucar D., Kaksonen A.H. (2011). Sulfidogenic fluidized bed treatment of real acid mine drainage water. Bioresour. Technol..

[16] Pagnanelli F., Viggi C.C., Cibati A., Uccelletti D., Toro L., Palleschi C. (2012). Biotreatment of Cr (VI) contaminated waters by sulphate reducing bacteria fed with ethanol. J. Hazard. Mater..

[17] Luptakova A., Macingova E. (2012). Alternative substrates of bacterial sulphate reduction suitable for the biological-chemical treatment of acid mine drainage. Acta Montan. Slovaca.

[18] Zhao Y., Ren N., Wang A. (2008). Contributions of fermentative acidogenic bacteria and sulfate-reducing bacteria to lactate degradation and sulfate reduction. Chemosphere.

[19] Waybrant K., Blowes D., Ptacek C. (1998). Selection of reactive mixtures for use in permeable reactive walls for treatment of mine drainage. Environ. Sci. Technol..

[20] Dvorak D.H., Hedin R.S., Edenborn H.M., McIntire P.E. (1992). Treatment of metal-contaminated water using bacterial sulfate reduction: Results from pilot-scale reactors. Biotechnol. Bioeng..

[21] Kijjanapanich P., Pakdeerattanamint K., Lens P., Annachhatre A. (2012). Organic substrates as electron donors in permeable reactive barriers for removal of heavy metals from acid mine drainage. Environ. Technol..

[22] Aoyagi T., Hamai T., Hori T., Sato Y., Kobayashi M., Sato Y., Inaba T., Ogata A., Habe H., Sakata T. (2017). Hydraulic retention time and pH affect the performance and microbial communities of passive bioreactors for treatment of acid mine drainage. AMB Express.

[23] Hao O.J., Chen J.M., Huang L., Buglass R.L. (1996). Sulfate-reducing bacteria. Crit. Rev. Environ. Sci. Technol..

[24] Nagpal S., Chuichulcherm S., Livingston A., Peeva L. (2000). Ethanol utilization by sulfate-reducing bacteria: An experimental and modeling study. Biotechnol. Bioeng..

[25] Bernardez L.A., de Andrade Lima L.R.P., de Jesus E.B., Ramos C.L.S., Almeida P.F. (2013). A kinetic study on bacterial sulfate reduction. Bioprocess Biosyst. Eng..

[26] Waybrant K., Ptacek C., Blowes D. (2002). Treatment of mine drainage using permeable reactive barriers: Column experiments. Environ. Sci. Technol..

[27] Zagury G.J., Kulnieks V.I., Neculita C.M. (2006). Characterization and reactivity assessment of organic substrates for sulphate-reducing bacteria in acid mine drainage treatment. Chemosphere.

[28] Kaksonen A.H., Franzmann P.D., Puhakka J.A. (2003). Performance and ethanol oxidation kinetics of a sulfate-reducing fluidized-bed reactor treating acidic metal-containing wastewater. Biodegradation.

[29] Reese B.K., Finneran D.W., Mills H.J. (2011). Examination and refinement of the determination of aqueous hydrogen sulfide by the methylene blue method. Aquat. Geochem..

[30] Strosnider W.H.J., Nairn R.W., Peer R.A.M., Winfrey B.K. (2013). Passive co-treatment of Zn-rich acid mine drainage and raw municipal wastewater. J. Geochem. Explor..

[31] Masindi V., Foteinis S., Chatzisymeon E. (2022). Co-treatment of acid mine drainage and municipal wastewater effluents: Emphasis on the fate and partitioning of chemical contaminants. J. Hazard. Mater..

[32] Parkhurst D.L., Appelo C.A.J. (2013). Description of Input and Examples for PHREEQC Version 3—A Computer Program for Speciation, Batch-Reaction, One-Dimensional Transport, and Inverse Geochemical Calculations.

[33] Ravel B., Newville M. (2005). Athena, Artemis, Hephaestus: Data analysis for X-ray absorption spectroscopy using IFEFFIT. J. Synchrotron Radiat..

[34] Sato Y., Hamai T., Hori T., Aoyagi T., Inaba T., Hayashi K., Kobayashi M., Sakata T., Habe H. (2022). Optimal start-up conditions for the efficient treatment of acid mine drainage using sulfate-reducing bioreactors based on physicochemical and microbiome analysis. J. Hazard. Mater..

[35] Zhang Z., Zhang C., Yang Y., Zhang Z., Tang Y., Su P., Lin Z. (2022). A review of sulfate-reducing bacteria: Metabolism, influencing factors and application in wastewater treatment. J. Clean. Prod..

[36] Plummer L.N., Parkhurst T.M.L., Wigley D.L. (1978). The kinetics of calcite dissolution in CO_2_-water systems at 5–60 °C and 0.0–1.0 atm CO_2_. Am. J. Sci..

[37] Hwang S.K., Jho E.H. (2018). Heavy metal and sulfate removal from sulfate-rich synthetic mine drainages using sulfate reducing bacteria. Sci. Total Environ..

[38] Loteto L.D., Monge O., Martin A.R., Ochoa-Herrera V., Sierra-Alvarez R., Almendariz F.J. (2021). Effect of carbon source and metal toxicity for potential acid mine drainage (AMD) treatment with an anaerobic sludge using sulfate-reduction. Water Sci. Technol..

[39] Le Faou A., Rajagopal B., Daniels L., Fauque G. (1990). Thiosulfate, polythionates and elemental sulfur assimilation and reduction in the bacterial world. FEMS Microbiol. Rev..

[40] Kiran M.G., Pakshirajan K., Das G. (2017). Heavy metal removal from multicomponent system by sulfate reducing bacteria: Mechanism and cell surface characterization. J. Hazard. Mater..

[41] Sun R., Li Y., Lin N., Ou C., Wang X., Zhang L., Jiang F. (2020). Removal of heavy metals using a novel sulfidogenic AMD treatment system with sulfur reduction: Configuration, performance, critical parameters and economic analysis. Environ. Int..

[42] Suzuki K., Kato T., Fuchida S., Tokoro C. (2020). Removal mechanisms of cadmium by δ-MnO_2_ in adsorption and coprecipitation processes at pH 6. Chem. Geol..

