# Supplementary material for: Geochemical Modeling of Heavy Metal Removal from Acid Mine Drainage in an Ethanol-Supplemented Sulfate-Reducing Column Test
Source: Materials (Basel). 2023 Jan 18;16(3):928. doi: 10.3390/ma16030928 (PMC9917845; doi:10.3390/ma16030928)
Supplement: Supplementary file 1 [file materials-16-00928-s001.zip › materials-2154952-supplementary.pdf]

Materials Special issue (New Trends of Functional Materials for Wastewater Treatment Applications)

Geochemical Modeling of Heavy Metal Removal from Acid Mine Drainage in an Ethanol-Supplemented Sulfate-Reducing Column Test

Keishi Oyama [1], Kentaro Hayashi [2], Yusei Masaki [2], Takaya Hamai [2], Shigeshi Fuchida [1,3], Yutaro Takaya [1,4] and Chiharu Tokoro [1,4,*]

[1] Faculty of Science and Engineering, Waseda University, 3-4-1 Okubo, Shinjuku-ku, Tokyo 169-8555, Japan
[2] Japan Organization for Metals and Energy Security (JOGMEC), 2-10-1 Toranomon, Minato-ku, Tokyo 105-0001, Japan
[3] Department of Marine Resources and Energy, Tokyo University of Marine Science and Technology, 4-5-7 Konan, Minato-ku, Tokyo 108-8477, Japan
[4] Faculty of Engineering, The University of Tokyo, 7-3-1 Hongo, Bunkyo-ku, Tokyo 113-8656, Japan
* Correspondence: tokoro@waseda.jp; Tel.: +81-3-5286-3320

1. Supplemental Data

1.1 Parameters Used for Geochemical Modeling

Table S1. Elements and Ionic species considered in the geochemical modeling.

Element	Ionic species
Al	$Al(OH)_4^-$, $Al(OH)_3$, $Al(OH)_2^+$, $AlOH^{2+}$, $AlSO_4^+$, Al^{3+}, $As(SO_4)_2^-$
C	HCO_3^-, H_2CO_3, CO_3^{2-}, CH_3COO^-, C_2H_5OH
Ca	Ca^{2+}, $CaSO_4$, $CaHCO_3^+$, $Ca(CH_3COO)^+$, $CaCO_3$, $CaHPO_4$, $CaOH^+$, $CaPO_4^-$, $CaNH_3^{2+}$, $CaH_2PO_4^+$, $Ca(NH_3)_2^{2+}$, $CaNO_3^+$
Cd	Cd^{2+}, $CdSO_4$, $CdCO_3$, $Cd(SO_4)_2^{2-}$, $CdOH^+$, $CdHCO_3^+$, $Cd(CO_3)_2^{2-}$, $Cd(OH)_2$, Cd_2OH^{3+}, $Cd(OH)_3^-$, $Cd(OH)_4^{2-}$, $CdNO_3^+$, $Cd(NO_3)_2$, $Cd(CH_3COO)^+$, $Cd(CH_3COO)_2$, $Cd(HS)_2$, $CdHS^+$, $Cd(HS)_3^-$, $Cd(HS)_4^{2-}$
Cu	$CuCO_3$, Cu^{2+}, $Cu(OH)_2$, $CuSO_4$, $Cu_2(OH)_2^{2+}$, $Cu(CO_3)_2^{2-}$, $CuHCO_3^+$, $CuNH_3^{2+}$, $Cu(OH)_3^-$, $Cu(OH)_4^{2-}$, $CuNO_2^+$, $CuNO_3^+$, $Cu(NO_2)_2$, $Cu(NO_3)_2$, $Cu(HS)_3^-$, $Cu(CH_3COO)^+$, $Cu(CH_3COO)^2$, $Cu(CH_3COO)_3^-$
Fe	Fe^{2+}, $FeSO_4$, $FeOH^+$, $FeHCO_3^+$, $FeHPO_4$, $Fe(OH)_2$, $FeH_2PO_4^+$, $Fe(OH)_3^-$, $Fe(HS)_2$, $Fe(HS)_3^-$, $Fe(CH_3COO)^+$, $Fe(OH)_2^+$, $Fe(OH)_3$, $Fe(OH)_4^-$, $FeOH^{2+}$, $FeHPO_4^+$, $FeSO_4^+$, $Fe(CH_3COO)^{2+}$, $Fe(CH_3COO)_2^+$, Fe^{3+}, $Fe(SO_4)_2^-$, $Fe(CH_3COO)_3$, $FeH_2PO_4^{2+}$, $FeNO_3^{2+}$, $Fe_2(OH)_2^{4+}$, $Fe_3(OH)_4^{5+}$
Mg	Mg^{2+}, $MgSO_4$, $MgHCO_3^+$, $MgCO_3$, $MgOH^+$, $MgHPO_4$, $MgH_2PO_4^+$, $MgPO_4^-$, $Mg(CH_3COO)^+$
Mn	Mn^{2+}, $MnSO_4$, $MnHCO_3^+$, $MnOH^+$, $Mn(OH)_3^-$, $Mn(OH)_4^{2-}$, $MnNO_3^+$, $Mn(NO_3)_2$, $Mn(CH_3COO)^+$, Mn^{3+}, MnO_4^{2-}, MnO_4^-
N	NH_4^+, NH_3, $NH_4SO_4^-$, NO_2^-, NO_3^-,
Na	Na^+, $NaSO_4^-$, $NaHCO_3$, $NaCO_3^-$, $NaHPO_4^-$, $Na(CH_3COO)$
P	HPO_4^{2-}, $H_2PO_4^-$, PO_4^{3-}, H_3PO_4
Si	H_4SiO_4, $H_3SiO_4^-$, $H_2SiO_4^{2-}$
S	H_2S, HS^-, S^{2-}, SO_4^{2-}, HSO_4^-
Zn	Zn^{2+}, $ZnSO_4$, $ZnCO_3$, $ZnOH^+$, $Zn(OH)_2$, $ZnHCO_3^+$, $Zn(SO_4)_2^{2-}$, $Zn(OH)_3^-$, $Zn(OH)_4^{2-}$, $ZnNO_3^+$, $Zn(NO_3)^2$, $ZnS(HS)^-$, $Zn(HS)_2$, $Zn(HS)_3^-$, $ZnS(HS)_2^{2-}$, $Zn(HS)_4^{2-}$, $Zn(CH_3COO)^+$, $Zn(CH_3COO)_2$

Table S2. Precipitates species considered in the geochemical modeling.

Precipitates	Chemical Equation	Log K
$CaCO_3$ (Calcite)	$CaCO_3 = Ca^{2+} + CO_3^{2-}$	-8.48
$CaSO_4 \cdot 2H_2O$ (Gypsum)	$CaSO_4 \cdot 2H_2O = Ca^{2+} + SO_4^{2-} + 2H_2O$	-4.61
$MgCO_3$ (Magnesite)	$MgCO_3 = Mg^{2+} + CO_3^{2-} + 3H_2O$	-7.46
$Cu(OH)_2$	$Cu(OH)_2 + 2H^+ = Cu^{2+} + 2H_2O$	8.67
CuS (Covellite)	$CuS + H^+ = Cu^{2+} + HS^-$	-22.3
$CuCO_3$	$CuCO_3 = Cu^{2+} + CO_3^{2-}$	-11.5
$Cu_3(OH)_2(CO_3)_2$ (Azurite)	$Cu_3(OH)_2(CO_3)_2 + 2H^+ = 3Cu^{2+} + 2H_2O + 2CO_3^{2-}$	-16.9
$Cu_2(OH)_2CO_3$ (Malachite)	$Cu_2(OH)_2CO_3 + 2H^+ = 2Cu^{2+} + 2H_2O + CO_3^{2-}$	-5.30
$Cd(OH)_2$	$Cd^{2+} + 2H_2O = Cd(OH)_2 + 2H^+$	13.6
CdS (Greenockite)	$CdS + H^+ = Cd^{2+} + HS^-$	-14.4
$CdCO_3$ (Otavite)	$CdCO_3 = Cd^{2+} + CO_3^{2-}$	-12.0
$CdSiO_3$	$CdSiO_3 + 2H^+ + H_2O = Cd^{2+} + H_4SiO_4$	9.06
$Zn(OH)_2$	$Zn(OH)_2 + 2H^+ = Zn^{2+} + 2H_2O$	12.2
ZnS (Sphalerite)	$ZnS + H^+ = Zn^{2+} + HS^-$	-11.5
$ZnCO_3$ (Smithsonite)	$ZnCO_3 = Zn^{2+} + CO_3^{2-}$	-10.0
$ZnCO_3 \cdot 1H_2O$	$ZnCO_3 \cdot 1H_2O = Zn^{2+} + CO_3^{2-} + H_2O$	-10.3

Table S3. Physical parameters used for the one-dimensional advection calculation.

Number of cells [-]	80
Length of one cell [m]	0.01
Total length [m]	0.8
HRT [sec]	90000
Flow velocity	8.9×10^{-6}
Flow direction	forward
Time step [sec]	1125

1.2 Quantification of Silicate Dissolution from Rice Husk (Abiotic Preliminary Kinetic Test)

Silicate dissolution from rice husk was evaluated by suspending 4.5 g of rice husk in 150 mL of distilled water for 9 hours. The solution sample was routinely taken to monitor pH and then filtered to measure the concentration of silicate by ICP-OES (Agilent 5110 ICP-OES, Agilent Technologies Inc.). Based on the dissolved silicate concentration as a function of time, a kinetic equation was constructed to simulate its dissolution behavior (Fig. S1). The constructed kinetic equation (Eq. S1) was then incorporated into the geochemical model calculation as explained in chapter 2.3.

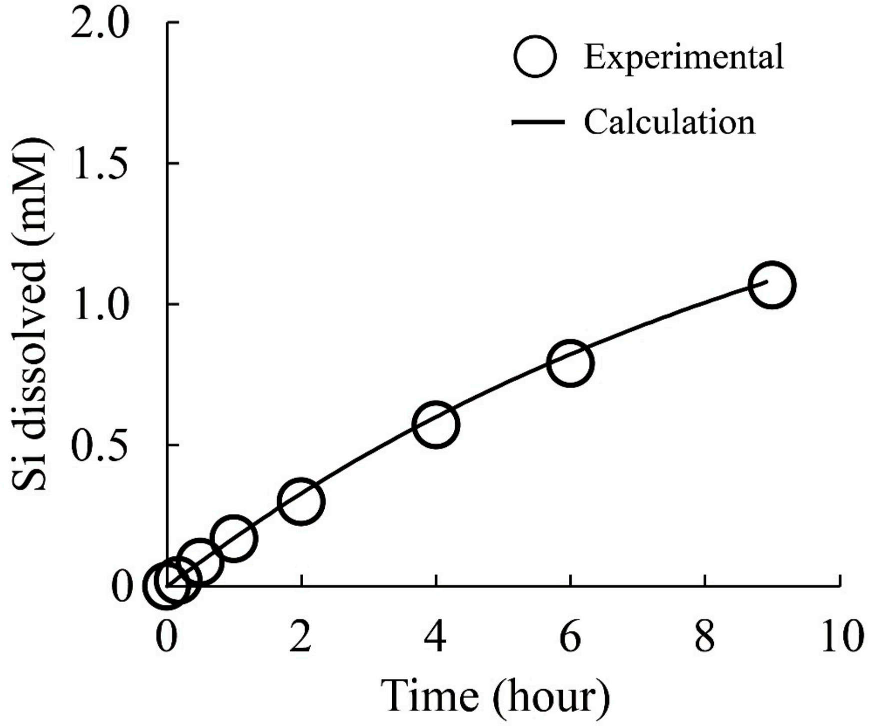

Figure S1. Changes in the concentration of Si dissolved from the rice husk with time (open circle). A solid line indicates the fitting curve calculated by the kinetic equation (Eq. S1).

$$d[\mathrm{Si}]/dt = k_6(1\text{-}10^{\mathrm{SI[SiO2]}}) \quad (k_6 = 5\times10^{-8}) \qquad \text{(S1)}$$

Where [Si] indicates the concentration of Si (mg/L), k_6 indicates the kinetic constant (s^{-1}), and SI[SiO_2] indicates the saturation index of SiO_2. The kinetic constant k_6 was a fitting parameter via the numerical fitting of the kinetic equation to the experimental result.
